# Influence of Porous Dressings Based on Butyric-Acetic Chitin Co-Polymer on Biological Processes In Vitro and In Vivo

**DOI:** 10.3390/ma12060970

**Published:** 2019-03-23

**Authors:** Witold Sujka, Zbigniew Draczynski, Beata Kolesinska, Ilona Latanska, Zenon Jastrzebski, Zbigniew Rybak, Boguslawa Zywicka

**Affiliations:** 1 Tricomed S.A., Lodz, Świetojańska Street 5/9, 93-493 Lodz, Poland; Ilona.Latanska@tricomed.com; 2 Department of Material and Commodity Sciences and Textile Metrology, Lodz University of Technology, Żeromskiego Street 116, 90-924 Lodz, Poland; zbigniew.draczynski@p.lodz.pl; 3 Institute of Organic Chemistry, Lodz University of Technology, Żeromskiego 116, 90-924 Lodz, Poland; beata.kolesinska@p.lodz.pl; 4 National Medicines Institute, Chełmska Street 30/34, 00-725 Warsaw, Poland; z.jastrzebski@nil.gov.pl; 5 Department of Experimental Surgery and Biomaterial Research, Wroclaw Medical University, Bujwida Street 44, 50-368 Wroclaw, Poland; zbigniew.rybak@umed.wroc.pl (Z.R.); boguslawa.zywicka@umed.wroc.pl (B.Z.)

**Keywords:** chitosan dressings, porous materials, biodegradation, biocompatibility, dressing material safety

## Abstract

In spite of intensively conducted research allowing for the development of more and more advanced wound dressing materials, there is still a need for dressings that stimulate not only reparative and regenerative processes, but also have a positive effect on infected and/or difficult-to-heal wounds. Porous dressing materials based on butyric-acetic chitin co-polyester containing 90% of butyryl and 10% of acetyl groups (BAC 90/10) can also be included in the group mentioned above. Two types of dressings were obtained by the salt leaching method, i.e. a porous sponge Medisorb R and Medisorb Ag with an antibacterial additive. The aim of the study was to evaluate biological effects of porous Medisorb R and Medisorb Ag dressings under in vitro and in vivo conditions. In an in vitro biodegradation test, no mass loss of Medisorb R dressing was observed within 14 days of incubation in physiological fluids at 37 °C. However, on the basis of the FTIR (Fourier Transform Infrared Spectroscopy) tests, surface degradation of Medisorb R dressing was observed. Additionally, the antibacterial activity of the porous Medisorb Ag dressing containing microsilver as an antibacterial additive was confirmed. The in vivo studies included inflammatory activity, skin irritation and sensitisation tests, as well an assessment of local effect after contact with subcutaneous tissue up to 6 months and skin wounds up to 21 days. In the in vivo tests, the dressings exhibited neither effects of skin irritation nor sensitisation. Under macroscopic examination, in full thickness defects of subcutaneous tissue and skin, the dressings caused wound healing with no inflammation, undergoing the most gradual biodegradation between weeks 4 and 8, and the observed differences were statistically significant. In the histological assessment, a weakened, limited inflammatory process associated with degradation of the material has been observed. The process of skin wound healing under Medisorb R dressing in the early period was accelerated compared to that observed in the control group with a gauze dressing.

## 1. Introduction

Wound healing is a spontaneous process that can be hampered in the case of large, long-lasting and difficult-to-treat chronic wounds. In such cases, if the autologous skin transplants are not available, biopolymers are used, which show the ability to initiate and stimulate the appropriate process of skin and epidermis healing [1]. Skin wound healing is a complex, biological process involving four overlapping stages: the inflammatory phase, the migration phase, the proliferative phase and the maturation phase causing remodelling [2]. Among many factors which influence the wound healing process, an extracellular matrix (ECM) plays a key role in the organization and management of cell phenotype, adhesion, migration and proliferation. The ECM contains molecules synthesized by fibroblasts including proteoglycans such as chondroitin sulphate, keratin sulphate, heparin sulphate, and fibrous proteins such as laminin, collagen and elastin. Moreover, the ECM serves as a reservoir of growth factors, proteases, cytokines, chemokines, etc. [3,4]. Apart from formation of granulation tissue in dermis, wound epithelialization [5], during which the keratinocytes and fibroblasts migrate to the wound, is the key point in the wound healing process [6]. Taking into consideration the complexity of the wound healing process, it is necessary to search for new materials able to imitate various tissues in terms of their function. The most commonly used natural polymers are polysaccharides: starch, cellulose, chitin, alginate and proteins/polypeptides such as wool, gelatin, silk, linen and collagen [7,8,9,10,11,12]. The biopolymers presented below meet the biocompatibility requirements [13,14] and they can influence different biological systems in various ways, for example, tissue processes connected to wound healing, endothelium in contact with endovascular devices, target cells in the genes therapy and stem cells in bioreactors [15,16,17]. Modern technologies enable the production of porous biomaterials and improvement of natural biosynthetic systems for targeted applications. Porous structures that map tissue architecture are obtained using a variety of techniques, such as solvent casting, solid particle leaching, gas frothing, phase separation, electrospinning, porogen leaching, fibre mesh, fast prototyping and freeze drying by congealing, allowing for the preparation of various useful forms in various applications [18,19,20,21]. Taking into consideration the stable and homogeneous porous structure, the possibility of modulating the pore sizes, enlarged surface and porous structure properties, it seems natural to seek new manufacturing methods of new porous materials, as well as to conduct research on the development of porous structures from various biopolymers [22]. Porosity of the biopolymer matrix is important in the healing process because it enables cell filtration and high permeability, as well as oxygen and nutrients diffusion [23]. One of biopolymers, widely used in biological tests, is chitin and its derivatives [24,25,26]. Chitin is non-toxic, biocompatible and does not evoke an allergic reaction; it has a positive impact on macrophage activation, increases the intensity of hypertrophic tissue granulation, growth of small blood vessels and expansion within the wound, which results in an accelerated process of wound healing [27]. Chitin is easily available; however, its main disadvantage is its almost complete insolubility in water and organic solvents. It demands modification in order to obtain derivatives with better utility properties [28,29]. One approach of chitin modification is based on preparing its ester derivatives, which are characterized by satisfactory solubility in organic solvents, leading to a possibility of using it in the production of various chitin ester-based materials [30,31,32,33,34,35]. The most commonly tested materials that stem from chitin are esters that contain acetyl groups or mixed esters. The best-known biological parameter in terms of wound healing is chitin derivative (DBC, dibutyryl-chitin), which contains both hydroxylic groups remnants of butyric acid [36]. Apart from DBC, chitin esters derived from aliphatic C3–C8 acids are also tested. Apart from classic chitin esters, the chitin co-polyesters are used, as well. There were attempts to obtain chitin co-polyester containing butyric groups and propionic and/or valeric acid residues, though they did not find application as a substrate for dressing production [37,38]. As a result of the performed tests, it was stated that a butyric-acetic chitin co-polymer containing 90% butyryl and 10% acetyl groups (BAC 90/10) [39] has properties that predispose it to the production of dressing materials. It is very soluble in organic solvents, which enables obtaining functional forms with maintaining fibre-forming properties. An effective method of producing this derivative on an industrial scale has been developed [40], which allowed for further research work on the production of materials useful in medicine. One of the function forms based on derivative BAC 90/10 is porous material. Its production on an industrial scale has been developed [41]. Porous materials derived from BAC 90/10 were obtained by the salt leaching method, which resulted in two formulations, Medisorb R material in the form of a porous sponge and Medisorb Ag, additionally equipped with a component with bacteriostatic/bactericidal activity. Product safety is the most important parameter that determines its usefulness aside from the required biological activity.

The aim of the presented research was the biological evaluation of the effect of Medisorb R and Medisorb Ag dressings in vitro and in vivo. As part of the in vitro study, it was planned to check the degradation of the Medisorb R dressing under the treatment with plasma/serum enzymes and bacteriostatic/bactericidal activity of the Medisorb Ag dressing. Safety and effectiveness of Medisorb R and Medisorb Ag dressings was planned to be checked in vivo in studies involving: the tests concerned toxic and irritation activity after multiple application on the skin, allergic activity, healing process in the subcutaneous tissue in the period of up to 6 months and dressing impact on the healing process of the full thickness skin loss in the period of 21 days.

## 2. Materials and Methods

### 2.1. Preparation of Medisorb R and Medisorb Ag Dressing Materials

Medisorb R and Medisorb Ag dressings [42,43] manufactured in laboratories in Tricomed S.A. (Lodz, Poland) company were tested in vitro and in vivo. The first stage in the manufacture of dressings in this form was the preparation of a homogeneous BAC 90/10 solution. Ethanol (10 L) was used to dissolve 250 g of BAC 90/10. The solution was poured (7 mL on one dressing with 9 cm diameter) on the surface on which a layer of porophor agent was regularly distributed (sodium chloride, particle diameter 0.16–0.4 mm, 10 g on one dressing with 9 cm diameter). After the solvent evaporation at a temperature of 40 °C, the dressing material was treated with deionised water. Salt removal was continued till the chloride ions disappeared in the washing media (the lack of chloride ions was confirmed by the absence of sediment precipitation against silver nitrate). After removing NaCl, the obtained dressing materials were dried at a temperature of 80 °C for 4 h.

Porous dressing enriched by the bactericide factor: The manufacturing process was analogical to the one described above with an additional phase of incorporation of the metallic silver on the surface in the form of micromolecules (microsilver) with bactericide characteristics confirmed. Silver was spread on the dressing material through spraying the silver suspension on the surface using a spray nozzle. The silver suspension was prepared by adding the metallic silver in powdered form of 1 g on 1 dm^3^ of water and then sonication of the blend was carried out using the ultrasound probe with 150 W power, frequency 40 kHz for 5–10 min, using a 2 cm diameter probe which was immersed in the solution 20 cm deep. Water excess was removed from the dressing in the drying process at 40 °C (at reduced pressure). The dressing materials, prepared in such a manner, were packed and submitted to the radiation sterilisation process in which the radiation dose was 25 kGy. The sterilisation process was validated according to the PN-EN ISO 11137-2 norm. It enabled obtaining materials which were characterised by high porosity up to 95–99% and chemical purity confirmed using organoleptic analysis, pH tests, permanganate oxidisability, absorbance in the ultraviolet radiation and the accurate conductivity of water extracts.

### 2.2. In Vitro Tests

The tests concerned the assessment of mass loss and possible products of decomposition of Medisorb R in plasma. Nine Medisorb R dressings of 10 cm diameter each were used. The dressing materials were divided into portions of the average mass of 20–60 mg. Next, the samples were placed in test tubes and were subjected to selected physiological fluid activity or water (control) and incubated with constant stirring (3 turns (360°)/min) for a given time at a temperature of 37 °C. After the elapsed time, the dressing samples were washed with water (6 cycles, 10 min each, with stirring (40 turns (360°)/min). After water removal, the samples were dried in a freeze-drier (0.1 mBa, 25 °C, 6 h) and the mass was measured. The average from three independent tests constituted the result. During the tests, the following devices were used: Metler Toledo (Columbus, OH, USA) XS105DU/M model analytical balance, SB3/Stuart^®^ rotator (Cole-Parmer, Stone, Staffordshire, UK), CLW 53 STD (POL-EKO-APARATURA Sp.J, Wodzislaw Slaski, Poland) incubator, Christ/Alpha 2-4 LSC freeze-drier (SciQuip LTD, Newtown, Wem, Shropshire, UK), and the following materials: sterile centrifuge tubes 15 mL (Axygen/28612055); sterile test tubes 1.5 mL (SARSTEDT/0000/2146101); test tubes for serum isolation (BD Vacutainer^®^/3119102) purchased by Sigma-Aldrich (Poznan, Poland). Table 1 presents the characteristics of the physiological fluids used. While choosing the physiological fluids, the effect of freezing on the activity of enzymes included in plasma was taken into consideration, as well as the manner of blood collection for plasma isolation (using EDTA-ethylenediaminetetraacetic acid). In order to establish the influence of the mentioned factors on test results, newly dissected blood plasma was used.

Chemical composition of Medisorb materials were proved using IR spectra tests. After each time of treatment, Medisorb samples were recorded using FTIR spectrometer System 2000 Perkin Elmer Cracow, Poland in form of thin porous films using an ATR (Attenuated Total Reflectance) adapter.

### 2.3. Examination of Antibacterial Properties of the Medisorb Ag Dressing

For tests, two types of dressings were used: Medisorb R (control) and Medisorb Ag, which in the final stage of production, was modified with microsilver in order to achieve the antibacterial activity. Tests on antibacterial activity were performed in compliance with the PN-EN ISO 20743:2013 norm. Six control samples and six samples with an antibacterial additive were used. The tested samples were placed in a separate sterile container. The samples were previously subjected to radiation sterilisation, so additional sterilisation was not necessary. The following bacterial strains were used in accordance with the PN-EN ISO 20743:20163 norm: *Staphylococcus aureus* ATCC 6538 and *Klebsiella pneumoniae* ATCC 4352. For each of the species, a suspension with a density from 1.0 × 10^5^ to 3.0 × 10^5^ CFU/mL was prepared. Tests with the use of each bacterial strain were conducted separately for each material. The test samples were treated with a bacterial suspension, where a 0.2 mL suspension was used for each sample. The suspension was pipetted on the sample on a couple of places. Directly after the application of bacterial suspension, 20 mL of neutraliser was added to the samples of control material and samples of Medisorb Ag. Six samples (three control samples of Medisorb R and tested samples of Medisorb Ag) were placed on a TSA (thermal sprayed aluminum coating) surface in dilution from 10^0^ to 10^−4^ in order to establish the starting bacteria quantity per sample. Six further samples (three control ones of Medisorb R and three test ones of Medisorb Ag) were incubated at a temperature of 37 ± 2 °C for 24 h. Twenty millilitres of neutraliser was added to the samples after incubation. Then, the samples were placed on a TSA surface in dilution from 10^0^ to 10^−4^ in order to establish the final bacterial quantity.

### 2.4. In Vivo Tests

• Skin irritation (repeated exposure)

For the test, three New Zealand White rabbits weighing 3.4–3.6 kg were used. The entire back and the flanks of the rabbits were depilated mechanically and cleaned with 70% ethanol. On the next day, two Medisorb R dressing samples with the dimensions of 2.5 × 2.5 cm, moistened with water to improve the contact with the skin, were applied to both flanks of each animal. The corresponding absorbent sterile gauze samples were used as the negative control. The samples were fixed using an occlusive compression dressing and remained in contact with the skin surface for 6 h. The same procedure was repeated over five consecutive days. After the last exposure, the skin reactions were evaluated at 1 h, 24 h, 48 h and 72 h based on the macroscopic assessment of erythema, eschar and oedema using a 0–4 grading scale.

• Skin sensitisation

The testing procedure for skin sensitisation using the Buehler method was carried out on male albino guinea pigs. Prior to starting the procedure, the back and flanks of the animals were depilated mechanically. Then, the Medisorb R dressing samples of 2.5 cm × 2.5 cm, moistened with water, were applied to the left flanks of ten animals, covered with an occlusive dressing and secured using elastic bandage wrapped around the trunks of the animals. The negative control samples of sterile absorbent gauze saturated with water were applied to the skin of five additional animals. Both the test samples and control samples remained in contact with the skin surface for over 6 h. This procedure was repeated for 3 days a week, for three consecutive weeks (induction phase). After a break of 14 days, the flanks of all animals were shaved and the samples were applied to the contralateral untreated flank of each animal (challenge phase). As in the induction phase, the samples were held in contact with the skin for 6 h under occlusive dressing. After 24 h and 48 h from the removal of the challenge samples, the skin reactions were evaluated based on the appearance of skin sensitisation symptoms, such as erythema or oedema.

• Test on the healing process in the subcutaneous tissue up to 6 months in the presence of Medisorb R dressing

Medisorb R sterile dressings of size 1.5 × 1.5 cm were used for the implantation research. The local effect of subcutaneous tissue was checked at week 1, 2, 4, 8, 16, 20 and 28 after dressing implantation in the subcutaneous tissue in the abdominal cavity of the rats. Tests at all examination points were made in the same manner.

Animal pre-operation stage: The research was done on 35 rats of the same inbreeding, Wistar, female, approximately 4–6 months old and 200 g in weight. The rats were kept under laboratory conditions and were given standard LSM (Lab Service Manager) feed and water ad libitum. The abdominal skin of the rats was depilated mechanically the day before the operation, and the size of the depilated surface was 7 × 5 cm. The animals were not fed 24 h before the treatment.

**Figure 1:**
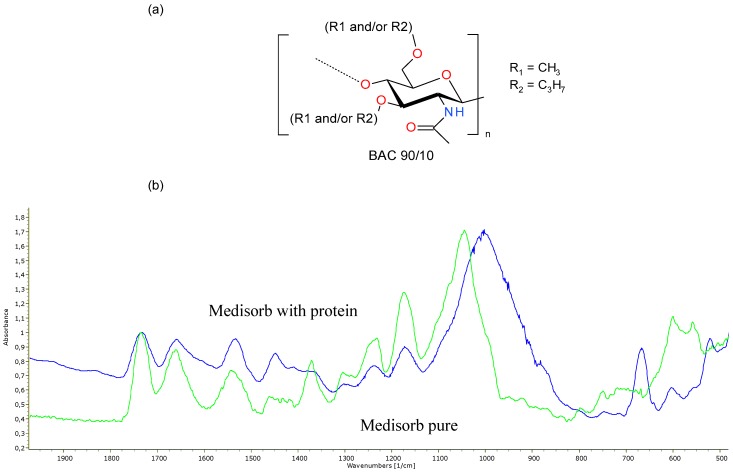
Panel (**a**): structure of BAC 90/10. Panel (**b**): FTIR ATR spectra of pure Medisorb (green) and Medisorb-protein (blue) complex after 336 h of treatment in fresh human plasma.

*Operation*: The process of dressing implantation was done under aseptic conditions. Rats were under the influence of general intraperitoneal anaesthetisation of Ketamine (Biowet, Pulawy, Poland) at a 25 mg/kg dose and Xylazine (Biowet, Pulawy, Poland) at a 5 mg/kg dose. The abdominal operational field was disinfected twice with SkinSept (Ecolab, Krakow, Poland). The 4 cm incision was made along the linea alba. After exposition of subcutaneous tissues and fascia of the abdominal wall, the pocket preparation was made in the subcutaneous tissue. Four pockets were made in each animal, two pockets on each side of the linea alba. Four implants (Figure 1) were put in these prepared pockets. Dressings were not fixed with threads because, being in contact with subcutaneous tissue, they adhered immediately. The skin wound was treated with single non-resorbable thread Nylon (PA) (Yavo Sp. z o.o., Belchatow, Poland). The skin was disinfected with SkinSept Pur or SkinSept Color (Ecolab Sp. z o.o., Krakow, Poland). After the operation, the animals were put in the lab cages, three females in each cage (see Figure S1 in Supporting Information).

*Autopsy tests*: Animal autopsies was made at week 1, 2, 4, 8, 12, 16, 20, 24 and 28 after the operation, taking into consideration three animals in the early stage and five animals in the later autopsy stages. The healing process of the operation wound was assessed during autopsy. After skin incision and preparation, the implants were visible. The assessment covered implant, tissues surrounding the implant, macroscopic changes in the peritoneum, liver condition, intestines, kidneys, lungs and heart. Photographic documentation was taken during autopsy. The fragments of the abdominal cavity cover altogether with the skin and implant were taken for histopathological tests.

*Microscopic tests*: Fragments of the abdominal cavity with the skin and implants were maintained for 48 h (at room temperature) in 10% water formaldehyde solution (earlier neutralised with calcium carbonate) in a phosphate buffer. After preliminary formalin maintenance, the tissue excess was removed from the samples. Next, the samples were dehydrated in acetone (at 56 °C), overexposed in xylene at room temperature and were put in paraffin blocks. The scraps of 4 µm width were cut on a rotation microtome Leica 2025 (Leica Microsystems, Werzlar, Germany). Prepared samples were coloured with haematoxylin and eosin (HE) using the Van Gieson method (VG) differentiating the connective tissue stroma, and then they were put in medium for submersion (CV Mount Medium, Leica Biosystems GmbH, Nussloch, Germany). Histopathological samples were assessed under an optical microscope (Olympus BX41, Olympus Corporation Tokyo, Japan) using various magnifications. Histopathological images were documented with the use of a software for picture analysis and acquisition (cellSens Software, [Ver.1.6] Imaging Software Olympus Cooperation, Tokyo, Japan). The inflammatory cells (granulocytes, lymphocytes, plasmatic tissues, macrophages, giant cells), necrosis presence, connective tissue growth, fibrous degeneration and fatty streak were taken into consideration in the tissue assessment.

• Test on healing process in full thickness skin loss in the period of 21 days in the presence of Medisorb dressing

Medisorb R dressings (size 2.5 × 2.5 cm) were used to evaluate their influence on the wound healing process. The gauze dressings of the same size were used as a control material. The research was run on New Zealand rabbits of both sexes and approximate weight of 2.7 kg (±200 g), which came from inbreeding at the Nofer Institute of Occupational Medicine in Lodz. Experimental animals were brought to the lab 1 month before the tests. The animals were kept in single cages and under controlled conditions regarding humidity (28–37%) and temperature (16–20 °C). The animals had unlimited access to water and were fed with standard granulated feed for rabbits (produced by Wytwornia Koncentratow i Mieszanek Paszowych, Motycz, Poland). The average use of feed per rabbit was 50–70 g. The test was run on 18 rabbits, divided into groups of three rabbits for each planned research date.

*Animal preparation for operation*: Rabbits were on a starvation diet 24 h before the planned procedure, though they had water access.

*Operation*: The rabbits were anaesthetised via intramuscular injection of Xylazine (Biowet, Pulawy, Poland) at a dose of 0.5 mg/kg and Ketamine (Biowet, Pulawy, Poland) at a dose of 5 mg/kg. The condition of full analgesia was obtained 10–15 min after addition of pharmaceuticals and lasted for 60–80 min. The full recovery lasted 120–140 min. After total analgesia was reached, the skin on both sides of the spine was disinfected with SkinSept Pur or SkinSept Color (Ecolab Sp. z o.o., Krakow, Poland). Afterwards, four operational, oval-shaped, full thickness skin wounds reaching fascia were made with a diameter of 2 cm. Two wounds on the right side of the spine were treated with Medisorb R, and two wounds on the left side were treated with the sterile control gauze dressing (Figure S2).

*Post-operative examination*: The animals were kept in separate cages with water and feed access. The animal condition, feed use, vet assessment concerning rabbit health and skin loss healing was monitored and assessed.

*Inspection, dressing change and wound observation*: Dressing of wounds was done every 24 h, in the same way as in the initial post-operative case. Medisorb R was not removed, but it was re-filled with a new layer up to the moment the wound was wet. The control gauze dressings were changed (Figure S3).

*Macroscopic and autopsy tests*: The photographic documentation was made and data concerning dressings and the process of wound healing was registered. The level of adherence, edge condition, ingrowth of new tissue, wound shriveling and scab formation was also monitored. In order to assess the difference between Medisorb R and control dressing, the wound diameter was measured. On the 3rd, 6th, 10th, 14th, 18th and 21st day after the operation, euthanasia was performed intravenously with pentobarbital (Morbital, Biowet Puland, Poland); the maximum dose was up to 80 mg/kg. The drug was given in fraction doses till the breathing and heartbeat stopped. Before the specimen was given, the general health condition of the rabbits was evaluated. During autopsy, the macroscopic wound image, presence and dressing was assessed. Skin fragments were taken for histopathological tests. The assessment of selected organs was also made.

*Microscopic tests*: Skin fragments for microscopic examination were prepared in the same way as described above.

*Statistics*: Data is presented as average. Also, the standard deviation of average quantities from measurements was determined on samples taken from n = 35 animals. The data was analysed with the ANOVA statistical test. Differences between averages were notified as significant with α = 0.05.

## 3. Results and Discussion

### 3.1. Test on Mass Loss and Possible Product of Degradation of Medisorb R in Plasma

While planning the tests and selection of physiological fluids, the following factors were under consideration: (a) influence of the plasma freezing on enzyme activity, and (b) method of blood collection for plasma isolation (with the use of EDTA). Assuming that the dissolution of Medisorb R should be correlated with enzymatic activity, the following physiological fluids were used:

1. frozen plasma (for checking the influence of freezing/de-freezing process and impact of EDTA for enzyme activity)
2. fresh plasma (sole effect of the presence of EDTA for enzyme activity)
3. fresh serum with full enzymatic activity.

Test results are presented in Table 2.

During the tests, it was found that even after 14 days of the treatment of Medisorb R dressing with all tested physiological fluids, no weight loss was observed. This can be connected with the fact that during incubation at a temperature of 37 °C, the enzymatic activity of the physiological fluid components was significantly reduced with time (finally, the protein denaturation took place, which resulted in plasma clouding). The observed changes in the sample mass could be the result of plasma and/or serum protein absorption on the dressing surface. This finding is consistent with the fact that the observed changes of the mass loss of Medisorb R were not dependent on the incubation time.

FTIR ATR analysis was made for samples of untreated Medisorb R dressings and material treated with fresh human plasma in order to test the dressing surface degradation and protein absorption on the dressing surface (Figure 1). Comparing spectra of samples treated with fresh human plasma and pure material, the decreasing of intensity of the vibration band of C=O at 1733 cm^−1^ in relation to the amide I signal at 1659 cm^−1^ was observed. It confirmed the sample surface degradation, which was connected to the hydrolysis of ester BAC 90/10 groups. The signals visible at 1445 cm^−1^ and 1002 cm^−1^ were connected with absorbed protein on the surface of dressing. An intense band at 667 cm^−1^ probably stemmed from the vibration of binding of phosphate derivatives.

### 3.2. Studies of Skin Irritation and Sensitisation Effects of the Medisorb R Dressing

The studies of skin irritation and sensitisation were conducted in accordance with the European Standard EN ISO 10993-10. Skin irritation in the form of erythema/eschar and oedema formation was scored according to the guideline-based scoring system depicted below (Table 3).

The scores were summed up and averaged independently for the test and control samples. The severity of the irritant reaction was calculated by subtracting the average score for the control from the average score for the test sample. The obtained value was the cumulative irritation index, allowing for classification of the skin irritation hazard (Table 4).

All animals appeared clinically normal throughout the study. There were no perceptible adverse local reactions following application of the tested product to the occluded rabbit skin. The calculated cumulative irritation index was “0”. Thus, the dressing can be classified as non-irritant. Since rabbit skin is more sensitive to irritant agents than human skin, the risk of local irritation from the Medisorb R dressing in humans should be regarded negligible.

Skin sensitisation reactions were graded according to the Magnusson and Klingman scale (Table 5). Grade 1 or more in the test group indicated sensitisation, provided a skin reaction in control animals was less than 1. Apart from daily clinical observation, the animals were weighed at the beginning of the experiment, then at weekly intervals and at the study termination.

Daily observation of the animals did not reveal any adverse clinical signs. There were no significant changes in body weight gains between the control and treatment group. Since after the challenge sample application, none of the animals presented erythematous or swelling responses, grade 0 was given to both animal groups. Based on this finding, it may be concluded that the Medisorb R dressing exhibited no sensitising potential in animal testing. Therefore, the risk of allergic reaction in humans can be considered negligible.

### 3.3. Local Evaluation of Subcutaneous Tissue Reaction after Medisorb R Implantation

**Figure 2:**
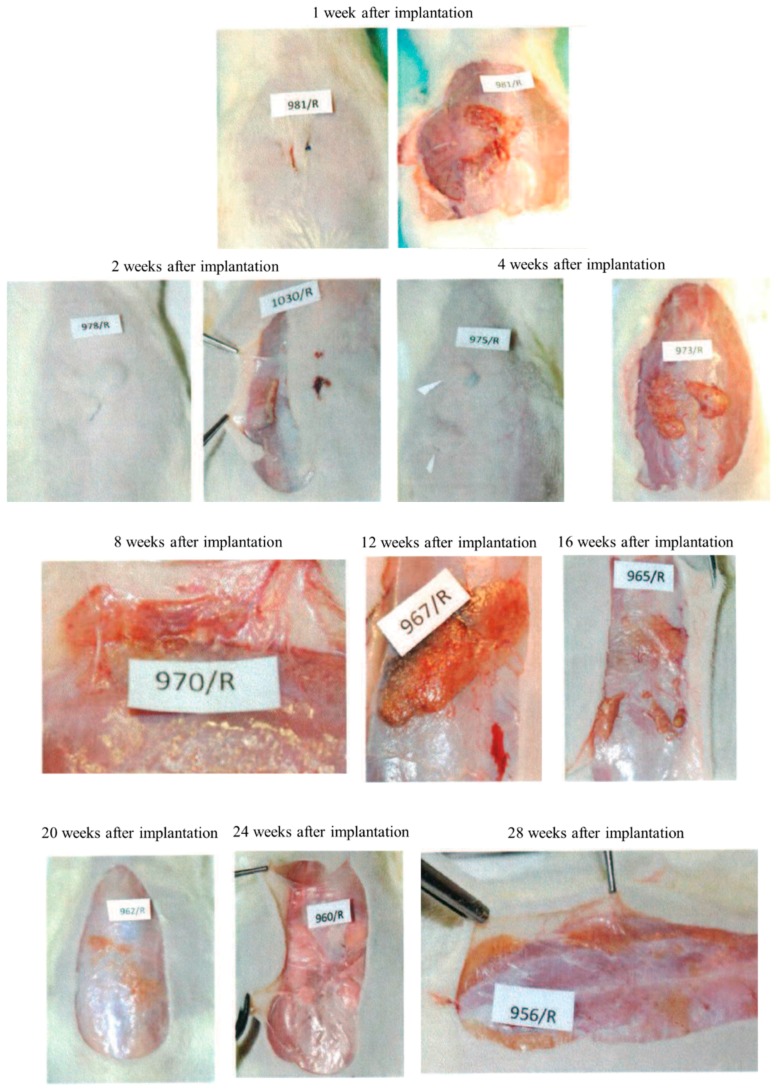
Macroscopic images after implantation of dressing Medisorb R in subcutaneous tissue within the 1st to 28th week.

All the animals survived till the moment of scheduled autopsy. During observation, all the animals had appropriate weight gain and appetite. Operational wounds healed through primary adhesion. During autopsy, no pathological changes in the peritoneum or organs were observed. The macroscopic evaluation was made at weeks 1, 2, 4, 8, 16, 20, 24 and 28 after Medisorb R dressing implantation in the subcutaneous tissue (Figure 2).

At week 1 after the implantation of the Medisorb R dressing on the surface of the skin in the abdominal area, the unhealed post-operative wounds were visible. In the place of subcutaneous dressing placement, the skin bulge of regular colour and look was visible. After the incision, the implants were found to be connected with the subcutaneous tissue structures. The tissues surrounding the implant were soft and had a regular look. The implant was covered with a tissue capsule with red hyperpigmentation through which the dressing was visible. After two weeks of the implantation, the implanted dressings were found to be covered with a tissue capsule connected to fascia and subcutaneous tissue. After four weeks of implantation, it was found that the tissue capsule surrounding the implants was covered slightly with blood vessels. After 8 weeks, it was observed that the implant was covered with a tissue capsule with blood vessels and was more flattened and more connected to the surrounding tissues, compared to the previous research term. Macroscopic images at weeks 12, 16, 20, 24 and 28 after implantation was similar. The skin surface, at the site of Medisorb R implantation, had a regular colour. The place of implant engraftment was either slightly visible or remained invisible. The implanted dressings were surrounded with a tissue capsule with thin blood vessels. They were connected to the subcutaneous tissue tightly and had a regular look. Over the course of time, the implants flattened and became smaller. They remained visible in the macroscopic images at week 28 after implantation.

#### Histological Tests

**Figure 3:**
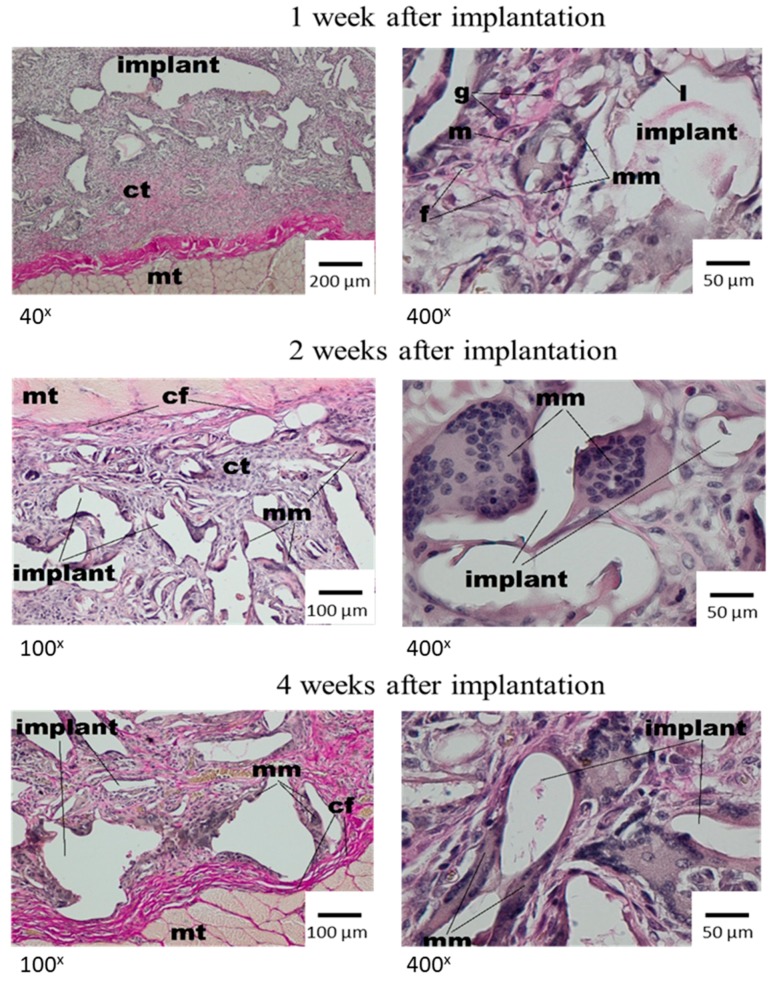
Microscopic examination after the implantation of Medisorb R dressing into the subcutaneous tissue of the rat; mt—muscle tissue, ct—connective tissue, l—lymphocyte, g—granulocyte, m—macrophage, mm—multinucleated macrophage. VG staining.

In the microscopic image, in the first week after dressing implantation, the irregular spaces were observed in the implant material. It was the result of the histopathological process of rinsing. Moreover, the fragments with loose connective tissue were observed, in which the presence of granulocytes, macrophages and a small amount of lymphocytes and fibroblasts was stated. In the direct implant neighbourhood, multinucleated macrophages containing elements of material were present (Figure 3).

**Figure 4:**
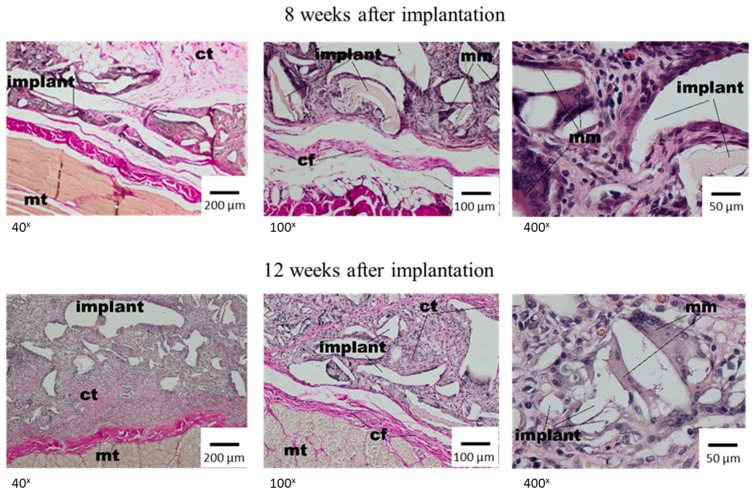
Microscopic image at week 8 and 12 after the implantation of Medisorb R into the subcutaneous tissues of the rat; mt—muscle tissue, ct—connective tissue, mm—multinucleated macrophage. VG staining.

After two weeks, the single irregular fibres and all implants were visible, and were surrounded with the band of connective tissue which separated them from subcutaneous tissue with a regular shape. The connective tissue had a fibrous shape from the side of the lateral muscles of the abdominal wall and connective tissue of the skin and loose structure rich in cells between the dressing fibres (Figure 3, middle panel). In the direct neighbourhood of the irregular implant fibres, the multinucleated macrophages were visible. At week 4 after implantation, the materials of irregular shape or spaces after rinsed material showed the connective tissue that had a fibrous shape with collagen fibres, fibroblasts, fibrocytes from the side of surrounding tissues, and loose connective tissue, rich in cells, from the material side. In the direct neighbourhood of the implant, the polymorphonuclear macrophages were visible. The subcutaneous tissue which surrounded the implant was unchanged (Figure 3, bottom panel). Eight weeks after the implantation of Medisorb R, the implant was smaller in comparison to previous research. It was separated with a band of connective tissue from surrounding muscles of the abdominal wall and from the layer of connective tissue of dermis, which at some places separated from the dressing (Figure 4, top panel). In the direct neighbourhood of smaller implant fibres, the connective tissue rich in cells, and polymorphonuclear macrophages were visible.

**Figure 5:**
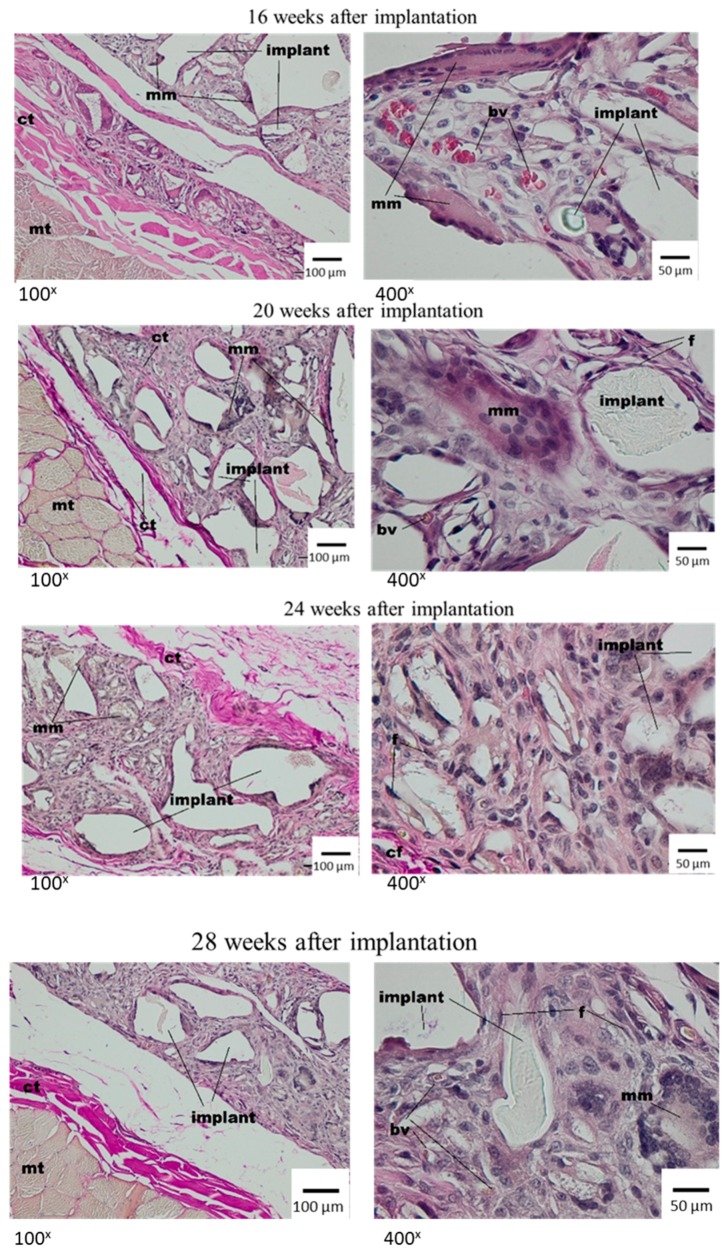
Microscopic image at weeks 16, 20, 24 and 28 after the implantation of Medisorb R into the subcutaneous tissue of the rat; ct—connective tissue, mm—multinucleated macrophage, mt—muscle tissue, bv—blood vessels. VG staining.

After 12 weeks of implantation, the histopathological image showed the subcutaneous tissue with a regular shape (Figure 4, bottom panel). The thin band of connective tissue separated the dressing from the surrounding tissues. The porous structure of the implant was filled with connective tissue, which was rich in cells from the material side and fibrous in between irregular fibres. The graded decrease of the implant was observed. After 16 weeks, the implants were separated from tissues with a thin layer of connective tissue. In between the dressing fibres, connective tissue rich in cells was visible, and the tissue had small blood vessels with inflammatory granulation; whereas in the direct neighbourhood of the material, giant cells were seen (Figure 5, top panel).

The histopathological image after 20 weeks of implantation was similar to data from previous tests. After 24 weeks of implantation, the bag surrounding the implant was built of thin connective tissues from the subcutaneous tissue and loose, rich in cells, connective tissue with polymorphonuclear macrophages around the dressing fibres (Figure 5, bottom panel).

**Figure 6:**
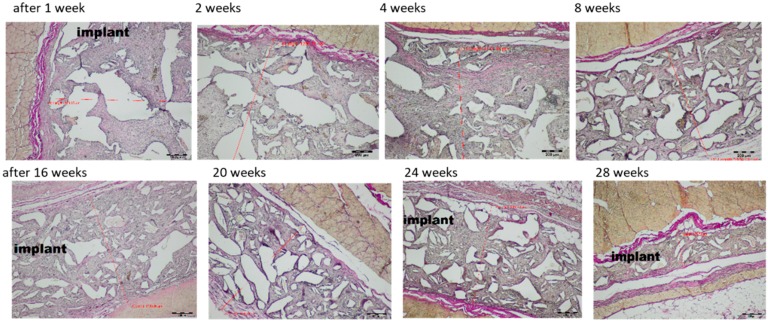
Microscopic image of the changes in width of Medisorb R dressing after implantation in the subcutaneous tissue in the rats, within 1–28 weeks. Magnifictaion: 40×.

**Figure 7:**
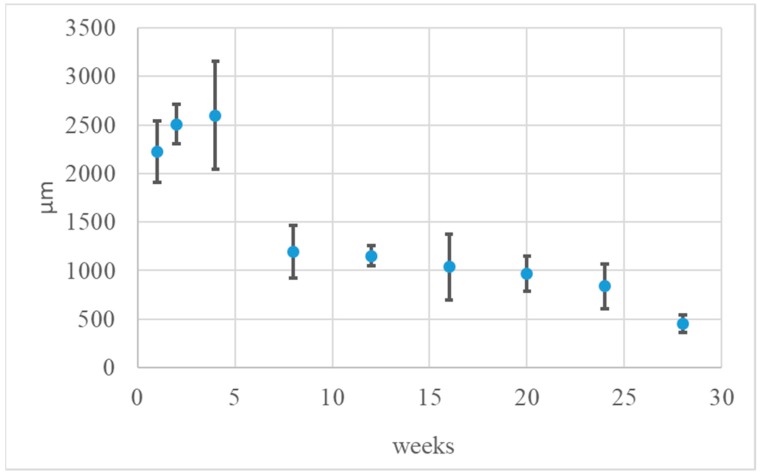
Graphical image of average Medisorb R dressing width at weeks 1, 2, 4, 8, 20, 24 and 28 after implantation into the subcutaneous tissue of the rat (n = 35).

After 28 weeks, there were smaller implants present in comparison to previous research terms. The implants were separated from the surrounding tissues with a thin band of fibrosis or steatosis (Figure 5 bottom panel). The process of implant degradation and healing did not finish after 28 weeks and in its neighbourhood, there was a loose connective tissue, rich in cells, with polymorphonuclear macrophages. Comparative microscopic image of the changes in width of tested dressing after implantation in the subcutaneous tissue within 1–28 weeks is presented in Figure 6, Table 6 and Figure 7.

Presented quantities are average values and ± SD; n—animal amount (35). The average width of the implant up to 4 weeks after implantation differed from the data observed from 8 to 28 weeks. The observed differences were statistically significant.

### 3.4. Wound Treatment of Full-Thickness Skin Loss with Medisorb R Dressing in Rabbits

In order to evaluate the influence of Medisorb dressing on the wound healing process, comparative macroscopic and histopathological tests were made on 3rd, 6th, 10th, 14th, 18th and 21st day after the operation in relation to wounds treated with gauze dressings [44]. The research was approved by the Local Commission of Ethics concerning Tests on Animals (opinion 39/2013). All the animals survived till the moment of scheduled autopsy. No clinical disease symptoms were observed. In macroscopic tests during autopsy, no pathological changes in the internal organs were found. One day after operation, Medisorb R and control dressings adhered to the wound edges and the skin. The Medisorb R dressing was not removed, and the change of controlled dressing was made after nourishing it in the physiological saline. The average wound size was 1.88 cm and 1.98 cm under Medisorb R and under the control dressing, respectively. The wound edges under the tested dressing were flat and dry, compared to the control dressing. Moreover, the wounds under Medisorb R shrunk and filled with a yellow and white membrane (Figure S4a). After the evaluation, a new Medisorb R dressing was applied, and the gauze dressing on the controlled wound was changed. Two days after operation, significant differences were observed in the wound image, depending on the dressing used. Medisorb R adhered to the wound edges on the whole wound surface, it was dry and covered with a brown scab. In the wound, there were yellow and white masses that moved to the centre of the wound from its edges and filled the bottom of the wound (Figure S4b). The average size of the wound under the Medisorb R dressing was 1.71 cm, whereas for the gauze dressing it was 1.81 cm. In the case of controlled wounds, they were filled with a thin, white and yellow membrane, or they were not filled. On the third day after surgery, the bottom of the wound with the test dressing was evenly covered with a scab, but the mass tissue did not reach the thickness of the skin (Figure S4c). The average wound sizes under the test and control dressing were 1.5 cm and 1.83 cm, respectively. The edges with the dressing were dry and flatter in comparison to the gauze dressing. Six days after the operation, in the wound treated with Medisorb R, epidermis was visible, starting from the edges. Tissue mass was localised under the scab. There were less newly formed tissues under the control dressing (Figure S4d).

On the 10th day of observation, the wounds under the Medisorb R dressing were covered with a dry scab and was a bit wider and harder compared to the control group. The edges of the wound were harder and shrunk, compared to wounds treated with the gauze dressing (Figure S4e). In the macroscopic evaluation, the wound healing process, which would need further wound treatment with test and control dressing, finished between the 10th and 14th days.

All wounds were shrunk and covered with the scab. In the wounds with no scab, a white scar was observed (Figure S4f). After 18 days post operation, all the wounds were healed. Spot scabs were visible. The wounds covered with Medisorb R were filled with pale scar tissue that was slightly wider in comparison to control group (Figure S4g). New, pink scar was visible on the 21st day once the scab fell out (Figure S4h). The research conclusion is that Medisorb R dressing adhered to wound and connected the edges with the bottom of the wound. The external integration with the scab was also observed. In the period of six days after operation, the tested material had a positive effect on the effusion phase, drying of the wound and simultaneous stimulation of growth phase. The process of epithelisation of skin loss took place under the scab from edges to the centre and at the early stage was faster with the use of Medisorb R dressing.

**Figure 8:**
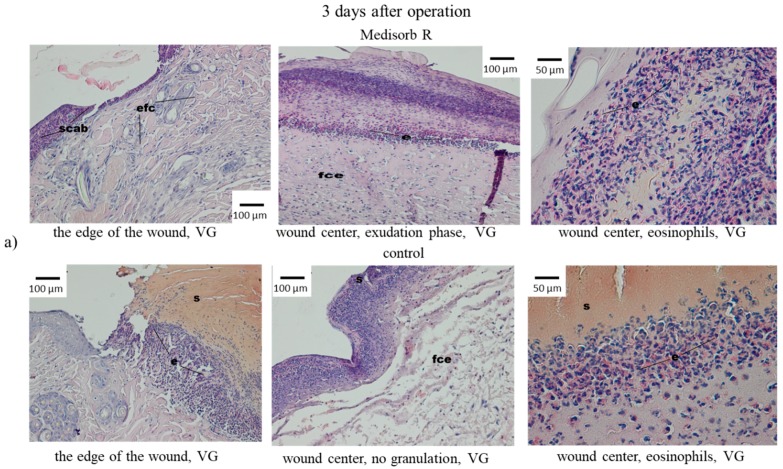

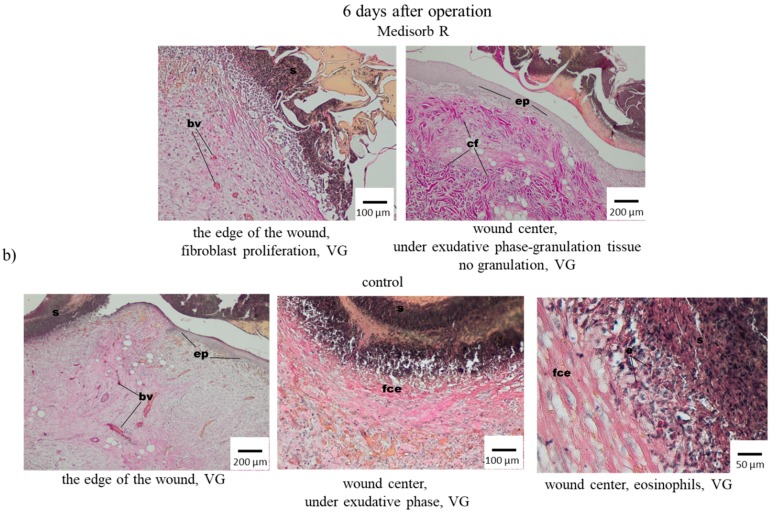
Microscopic images of skin wounds in rabbits, treated with Medisorb R or control gauze dressing after (**a**) 3 days and (**b**) 6 days of the operation; fce—fibre–cell effusion, s—scab, e—eosynophils, bv—blood vessels, ep—epitelum (epidermis), cf—colagen fibres.

Along with macroscopic tests, the microscopic (histopathological) tests of wounds treated with Medisorb R and gauze dressing as control dressing were run. In the microscopic tests, on the third day after operation, the wounds were covered with dressing and thin layer of homogenous mass, which were the image of a scab. Directly beneath them, in the centre of the wound, was the effusion of the fibre–cell with the advantage of eosinophilia. Short fragments of stratified epithelium were observed by the wound edges (Figure 8a). In the controlled wound, the bottom and centre of the wound was filled with fibre–cell effusion with dominant eosinophilia. Short fragments of newly-grown stratified epithelium were present at the wound edges (Figure 8).

On the sixth day after operation, in the case of wounds supplied with Medisorb R, the histopathological tests showed an in-growth of newly-formed epithelium that covered the fresh connective tissue under a homogenous mass and the remnants of fibre–cell effusion. Multiple fibroblasts and collagen fibres were observed under the newly-formed epithelium. The centre of the wound, which was not covered with epithelium, was filled with rich-in-cells connective tissue with blood vessels of granulation tissue character, in which many fibroblasts and eosinophils and less neutrophilia and lymphocytes were present (Figure 8b). However, in the case of the wounds treated with gauze dressings, loose subcutaneous tissue was visible, to which thin, newly-formed layer of granulated tissue with blood vessels was attached. The presence of thin layer of fibro-cell and homogenous mass was observed. Cellular blood elements were present in the effusion altogether with neutrophils. The new epidermis was observed on the wound edges which spread between the connective tissue and amorphous mass (Figure 8b).

**Figure 9:**
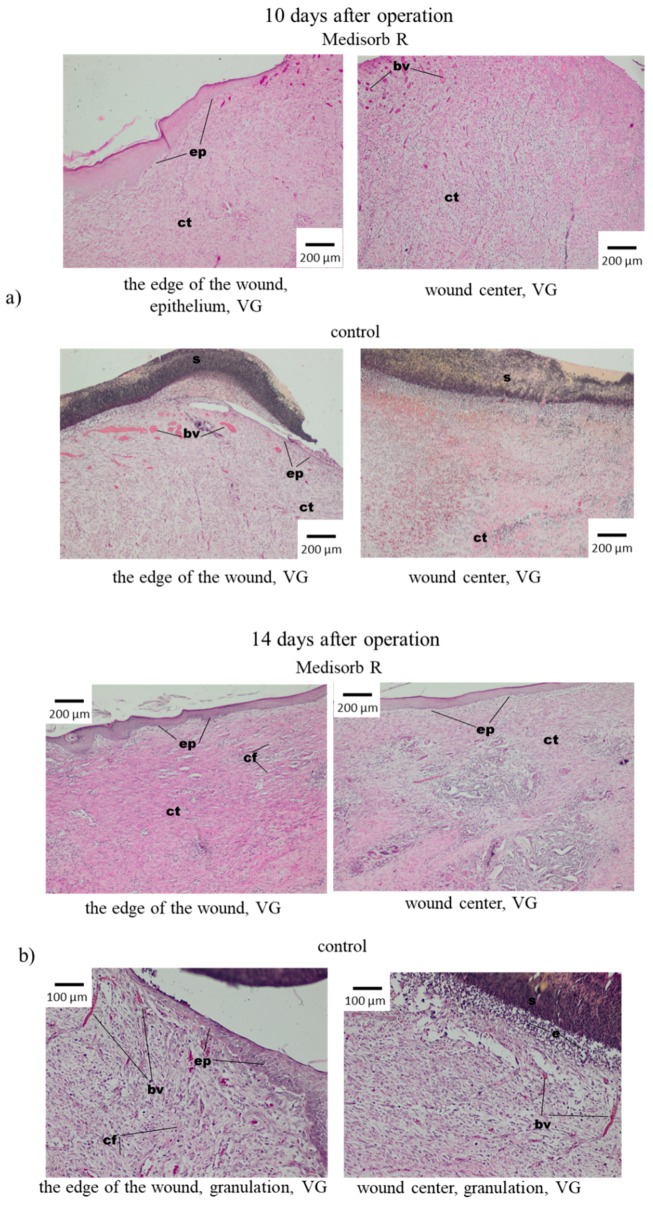
Microscopic images of skin wounds of the rabbits that were treated with Medisorb R or control gauze dressing after (**a**) 10 days and (**b**) 14 days of operation; e—eosynophils, bv—blood vessels, ep—epitelum (epidermis), cf—colagen fibres.

In controlled tests after 10 days from the operation, the loss was covered with a homogenous mass (scab). The epidermis reached the wound edge, between the connective tissue rich in cells and the homogenous mass. The epithelial surface was visible on a bigger surface in comparison to the previous research terms. In the wound centre, no fibre–cell effusion and granulation tissue were observed. At the bottom of the wound, the formation of connective tissue with collagen fibres was observed (Figure 9a).

**Figure 10:**
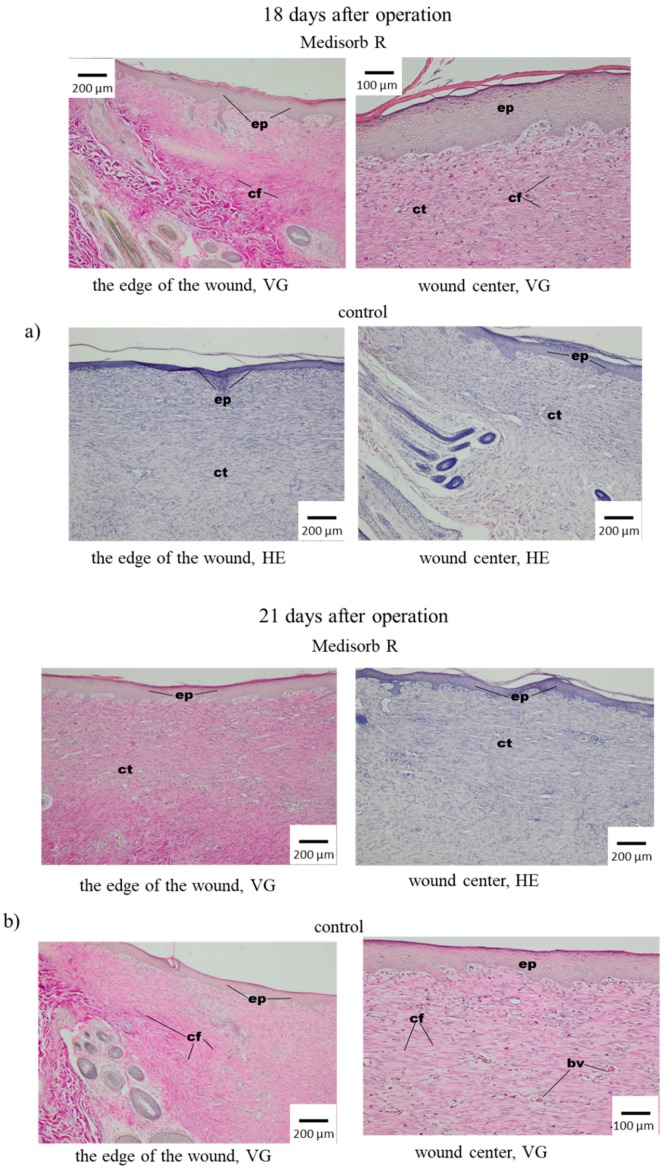
Microscopic images of skin wounds of the rabbits treated with Medisorb R dressing and control gauze dressing (**a**) 18 days and (**b**) 21 days after operation; bv—blood vessels, ep—epitelum (epidermis), cf—colagen fibres, ct—connective tissue.

On the 14th day, the wounds covered with Medisorb R showed the multiplication of fibrous connective tissue that contained numerous fibroblasts and collagen fibre, which almost completely covered the wide space of keratosis of the epithelium. In the centre of the wound, the epithelium was thinner. Some space of the wound was not covered with epithelium (Figure 9b). In control tests, on the 14th day after operation, it was found that the wounds were significantly smaller in comparison to the image after 10 days. It was observed that the epithelium grew from the edges to the centre of the wound, which filled the loss of connective tissue, which on the edges had a granulated structure and contained fibrocytes, fibroblasts and collagen fibres. In the centre of the wound, newly-grown tissue was not covered with epithelium. After 18 and 21 days from the operation, most wounds under Medisorb R were covered with epithelium with keratosis, in which glands and skin elements were built on the edges (Figure 10a). Similar images were observed for controlled wounds. At the bottom of the wounds, the connective tissue was visible, which was granulated and contained fibrocytes, fibroblasts and collagen fibres.

Twenty-one days after operation, the skin wounds were treated with Medisorb R dressing, and in most animals, the wounds were covered with a layer of epithelium, which was wider at the edges and thinner at the centre. Under the epithelium, connective tissue with collagen fibre was found, and less numerous fibrocytes were growing. In part of the wound, there was a homogenous mass with the remnants of effusion. In the case of the wounds that were treated with the control gauze dressing, on the 21st day after operation, the wound was filled with connective tissue, which was almost completely covered with epithelium with keratosis in which glands and skin elements were built. Newly-grown epithelium was wide at the edges and narrow down to the centre. In part of the preparation, the epithelium was covered with homogenous mass (scab), which was disconnected by migrating layer of epithelium. Under the newly-grown epithelium, the presence of connective tissue rich in cells was observed and contained eosinophils and less numerous lymphoid cells. In the histological examination, changes in the wound healing process using Medisorb R dressing and control one were stated. In the early stage, Medisorb R accelerated the epithelisation process, evoked less intense inflammatory effect with less intense induction of granulocytes and lowered the effusion phase and accelerated the creation phase.

### 3.5. Antibacterial Activity of the Medisorb Ag Dressing

The purpose of the test was to evaluate the antibacterial activity of Medisorb Ag in comparison to Medisorb R dressing, which was used as a reference sample. According to the criteria established in PN-EN ISO 20743:2013, if the antibacterial activity level in A is within the 2 ≤ A <3 range, then the tested material has a significant antibacterial activity, while if A > 3, the material is classified as strongly antibacterial. In the research on Medisorb Ag, the PN-EN ISO 20743:2013 requirements were met: (a) inoculum concentration was from 1 × 10^5^ to 3 × 10^5^; (b) the difference for three controlled samples, direct after inoculation was ≤1; (c) the value of growth F for controlled device was ≥1. Meeting the requirements allowed establishing conditions for marking the A value for Medisorb Ag (Table 7).

The result of antibacterial activity for Medisorb Ag was established at A = 2.83 for *S. aureus*, which indicated that the material had significant antibacterial activity. For *K. pneumoniae*, the value was A = 4.63, which indicated the strong antibacterial activity of the device.

Medisorb Ag was also submitted to in vivo tests, alike Medisorb R. It was confirmed that the dressing that contained silver did not show any cytotoxic allergic reaction. The dressing in the subcutaneous tissue underwent degradation and cramped. However, the small residue of dressing was found till the end of the observation phase (up to 28 weeks). The process of wound healing with the use of dressing evoked slight inflammation, which was connected to material degradation and led to filling the implant with connective tissues rich in cells. The dressing adhered to the wound and allowed for its edges to integrate with the bottom of the wound. The effusive phase was shortened and the creation phase was accelerated. The epithelisation phase under the dressing was shorter in resemblance to the control sample. On the basis of the tests it was stated that it was characterised by high biocompatibility.

The problem of difficult-to-heal wounds is trying to be solved with the use of target therapies or with the use of innovative dressings which show bioactivity features [45]. Unfortunately, difficult-to-heal wounds are usually infected by drug resistance bacteria, which hampers the healing and regeneration process [46]. As a result, Medisorb R dressing characterised using a sponge structure, based on resorbable derivative of butyric-acetic chitin co-polymer (BAC 90/10) and its version supplemented with silver ions (Medisorb Ag) was invented. In biocompatibility tests of Medisorb R on an animal model, no inflammatory effect was stated. Bioactivity tests of Medisorb R were made after its implantation in the subcutaneous tissue of the rats for 28 weeks. During autopsy, the dressing with surrounding bag was visible in the macroscopic image in an unchanged subcutaneous tissue at all test time points. In histological tests, the healing process evoked light and obstructed inflammatory effect which was connected with material degradation, and led to filling the implant with connective tissue rich in cells which had the feature of granulation tissue. In direct material neighbourhood, polymorphonuclear macrophages were visible. A gradual biodegradation and decreasing of implant size between weeks 1 and 28 29 of the experiment was stated. At the early stage, up to fourth week after implantation, the dressing width altogether with granulation tissue was higher and reached 2598.27 (±553.34). Since week 8, the average dressing width was smaller and at week 28, it was 453.39 (±88.25). The observed differences were statistically interchangeable. The obtained results indicate that up to the fourth week, there was significant growth of ECM within implant borders, then between weeks 4 and 8, there was a decrease in the average implant width, which was correlated with intense amount of polymorphonuclear macrophages, which were visible in the histological image. After that time, the average implants width decreased altogether with material amount. The degradation and healing processes were not finished after week 28, and in its direct neighbourhood, the connective tissue rich in cells, with polymorphonuclear macrophages, was visible. The tests of full thickness skin loss treatment showed that Medisorb R dressing had a more positive effect on reparation process of skin loss in comparison to the control gauze dressing. In the early stage, it accelerated the epithelisation process, it evoked slight inflammatory effect with delicate granulocytes induction (eosins connected to inflammation reaction), reduced the effusion phase and accelerated the creation phase. Similar histological image for the healing process was shown for the tests in which di-O-butyryl chitin (DBC) was used [47], and in which delicate inflammatory effect with macrophages presence, increase of vascularisation and then fibrosis was observed.

## 4. Conclusions

The conducted research proves that porous dressing materials Medisorb R and Medisorb Ag are safe materials used in wound healing process. Medisorb R undergoes slow degradation in human plasma, which was confirmed using FTIR tests, although in tests based on material mass determination in physiological fluids, no mass loss was observed. In animal studies, the dressing caused no skin irritation or sensitisation, so the risk of adverse local reactions in humans can be considered clinically insignificant.

The most important conclusions derived from the research on the healing process with the use of Medisorb R in subcutaneous tissue in the period of up to 6 months are as follows:

1. In all scheduled tests (up to week 28), Medisorb R dressing covered with thin bag was visible in macroscopic image in unchanged subcutaneous tissue.
2. Medisorb R dressing underwent degradation and gradual reduction in subcutaneous tissue.
3. The healing process of Medisorb R evoked little inflammation effect, which was connected to material degradation and led to fulfilling the implant with rich-in-cell tissue of granulation feature.
4. The process of degradation and Medisorb R dressing healing was not finished by week 28 after implantation, and in the material neighbourhood, rich-in-cell connective tissue with multinuclear macrophages was present,
5. Medisorb R dressing was characterised by high biocompatibility.

From the conducted research, due to the influence of Medisorb R on operational skin loss, it is stated that the tested material has a more positive effect on the repair process of skin loss in comparison to control material. In the early stage, it accelerated the epithelisation process, evoked less intense inflammatory effect with less effusion, and enhanced the creation phase.

The antibacterial activity of material Medisorb Ag with microsilver as an antibacterial additive was determined at A = 2.83 for *S. aureus*, which indicates significant bacterial activity. For *K. pneumoniae* the value was A = 4.63, which indicates high antibacterial activity of the product. The criteria of PN-EN ISO 20743:2016 were used in the test.

## Figures and Tables

**Table 1:** Characteristics of physiological fluids used in the studies.

Physiological Fluid	Source/Parameter
Frozen human plasma	Military Centre of Blood Donation and Blood Treatment in Wroclaw, Poland
Fresh human plasma	Regional Centre for Blood Donation and Blood Treatment in Wroclaw, Poland
Fresh human serum	Own preparation
High-purity water	System Direct-Q^®^ Millipore (Merck KGaA, Darmstadt, Germany)/18.2 MΩ cm

**Table 2:** The results regarding the mass loss of Medisorb R dressing under the influence of various physiological fluids.

**Incubation Time** **(Days)**	**Control** **Average Mass Loss ± SD (%)**	**Frozen Plasma** **Average Mass Loss ± SD (%)**
3	−4.0 ± 0.2	−2.5 ± 0.2
9	−4.6 ± 0.3	−2.0 ± 0.6
14	−4.6 ± 0.2	−1.9 ± 0.6
**Incubation Time** **(Days)**	**Control** **Average Mass Loss ± SD (%)**	**Fresh Plasma** **Average Mass Loss ± SD (%)**
3	−4.0 ± 0.2	−2.0 ± 0.2
9	−4.6 ± 0.3	−2.7 ± 0.6
14	−4.6 ± 0.2	−3.1 ± 0.5
**Incubation Time** **(Days)**	**Control** **Average Mass Loss ± SD (%)**	**Fresh Serum** **Average Mass Loss ± SD (%)**
3	−4.1 ± 1.9	−5.1 ± 0.1
9	−5.2 ± 2.0	−5.1 ± 0.3
14	−4.4±1.3	−4.6±0.1

**Table 3:** Scoring system of the skin irritation test.

Reactions	Score
**Erythema and Eschar**	
No erythema	0
Very slight erythema (barely perceptible)	1
Well-defined erythema	2
Moderate erythema	3
Severe erythema (beet redness) to eschar formation preventing grading of erythema	4
**Oedema**	
No oedema	0
Very slight oedema (barely perceptible)	1
Well-defined oedema (edges of area well-defined by defined raising)	2
Moderate oedema (raised approx. 1 mm)	3
Severe oedema (raised more than 1 mm and extending beyond exposure area)	4
**Maximum Possible Score for Irritation**	**8**

**Table 4:** Cumulative irritation index categories.

Average Score	0–0.4	0.5–1.9	2–4.9	5–8
Response Category	Negligible	Slight	Moderate	Severe

**Table 5:** Skin sensitisation reactions were graded according to the Magnusson and Klingman scale.

Skin Reaction	Grading Scale
No visible change	0
Discrete or patchy erythema	1
Moderate and confluent erythema	2
Intense erythema and/or swelling	3

**Table 6:** Average Medisorb R dressing width at weeks 1, 2, 4, 8, 20, 24 and 28 after implantation into the subcutaneous tissue of the rat (n = 35). and graphical image of the process.

Implantation Time (weeks)									
	1	2	4	8	12	16	20	24	28
Implant width (µm)	2222.88	2505.74	2598.27	1192.35	1152.94	1039.54	966.15	840.42	453.39
SD	±315.49	±203.96	±553.34	±273.45	±106.88	±338.94	±179.89	±230.58	±88.25

**Table 7:** Results of tests on antibacterial activity of Medisorb Ag material.

Value of Antibacterial Activity for Effusion Method		
Bacteria	*Staphylococcus aureus* ATCC 6538	*Klebsiella pneumonie* ATCC4352
Inoculum concentration	2.7 × 10^5^	1.0 × 10^5^
Value growth F (F = log C_T_ − log C_0_)	1.7	2.78
Value growth G (G = log T_T_ − log T_0_)	−1.13	−1.85
A value of antibacterial activity (A = F − G)	2.83	4.63

C_T_—bacteria number in the controlled sample after incubation; C_0_—bacteria number in the controlled sample before incubation; T_T_—bacteria number in the device sample with the antibacterial top after incubation, T_0_—bacteria number in the device sample with antibacterial top before incubation.

## Supplementary Materials

The following are available online at https://www.mdpi.com/1996-1944/12/6/970/s1, Figure S1: Intraoperative image. Medisorb R dressing implantation in the subcutaneous tissue in the rat abdominal cavity, Figure S2: Intraoperative image (top panel). Rabbit skin loss of full thickness. Initial post-operative wound healing: Medisorb (middle panel) and gauze control dressing (bottom panel), Figure S3: Wound supplying with Medisorb R and gauze control dressing (top panel). Wound supplying with the knitted band (bottom panel), Figure S4: Macroscopic images of operational skin loss in rabbits, 1, 2, 3,6, 10, 14, 18 and 21 days after operation. The wounds were treated with Medisorb R dressing and gauze dressing as control.
