# Supplementary material for: Influence of Porous Dressings Based on Butyric-Acetic Chitin Co-Polymer on Biological Processes In Vitro and In Vivo
Source: Materials (Basel). 2019 Mar 23;12(6):970. doi: 10.3390/ma12060970 (PMC6471426; doi:10.3390/ma12060970)
Supplement: Supplementary file 1 [file materials-12-00970-s001.pdf]

materials

Influence of porous dressings based on butyric-acetic chitin co-polymer on biological processes in vitro and in vivo

Witold Sujka [1,*], Zbigniew Draczynski [2], Beata Kolesinska [3], Ilona Latanska [1], Zenon Jastrzebski [4], Zbigniew Rybak [5] and Boguslawa Zywicka [5]

1 Tricomed S.A., Lodz, Świetojańska 5/9, 93-493 Lodz, Poland; Witold.Sujka@tricomed.com, Ilona.Latanska@tricomed.com
2 Department of Material and Commodity Sciences and Textile Metrology, Lodz University of Technology, Żeromskiego 116, 90-924 Lodz, Poland; zbigniew.draczynski@p.lodz.pl
3 Institute of Organic Chemistry, Lodz University of Technology, Żeromskiego 116, 90-924 Lodz, Poland; beata.kolesinska@p.lodz.pl
4 Narodowy Instytut Leków, Chełmska 30/34 00-725 Warsaw, Poland; z.jastrzebski@nil.gov.pl
5 Department of Experimental Surgery and Biomaterial Research, Wroclaw Medical University, Bujwida Street 44, 50-368 Wroclaw, Poland; zbigniew.rybak@umed.wroc.pl, boguslawa.zywicka@umed.wroc.pl
* Correspondence: Witold.Sujka@tricomed.com; Tel.: +48-42-689--6521

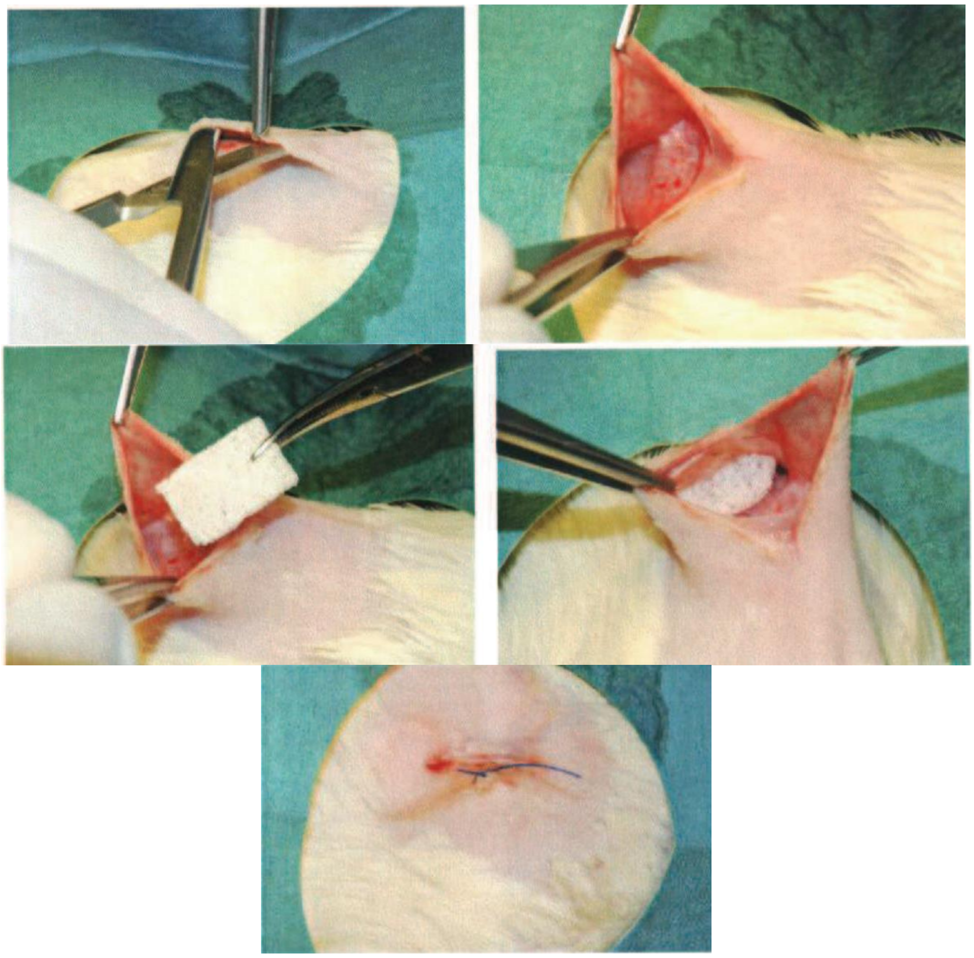

Figure S1. Intraoperative image. Medisorb R dressing implantation in the subcutaneous tissue in the rat abdominal cavity.

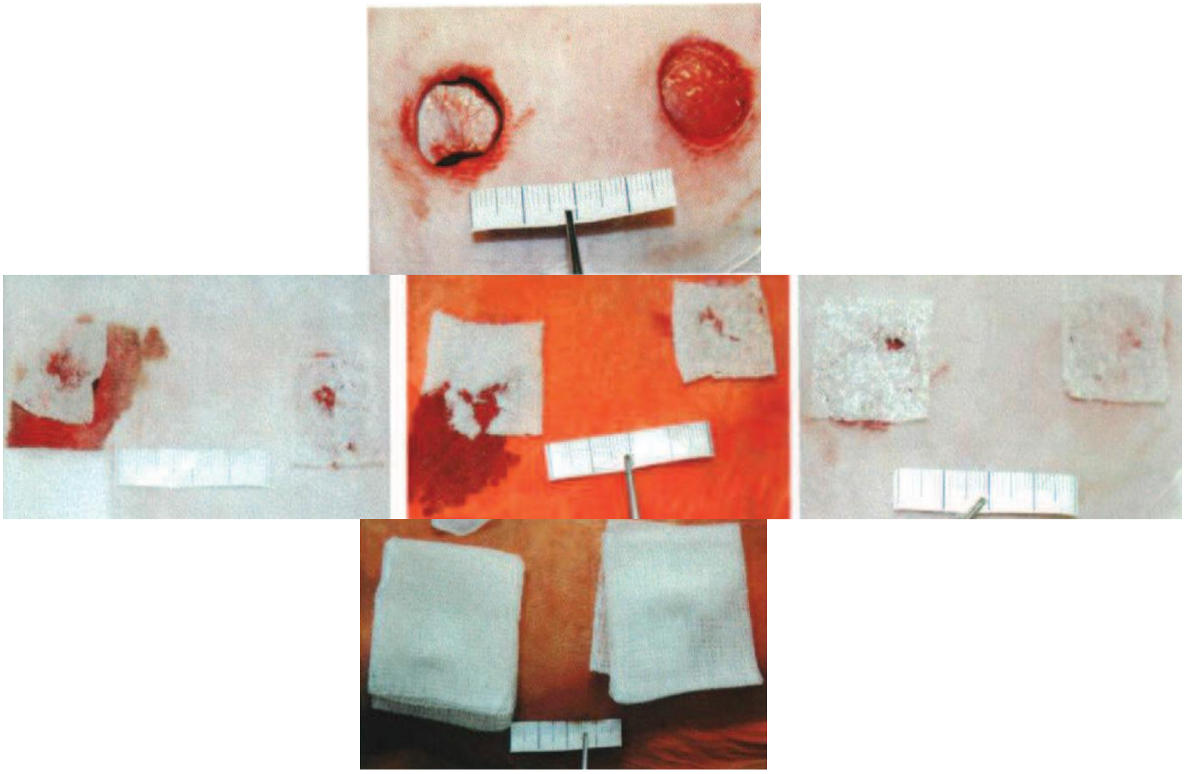

Figure S2. Intraoperative image (top panel). Rabbit skin loss of full thickness. Initial post-operative wound healing: Medisorb (middle panel) and gauze control dressing (bottom panel).

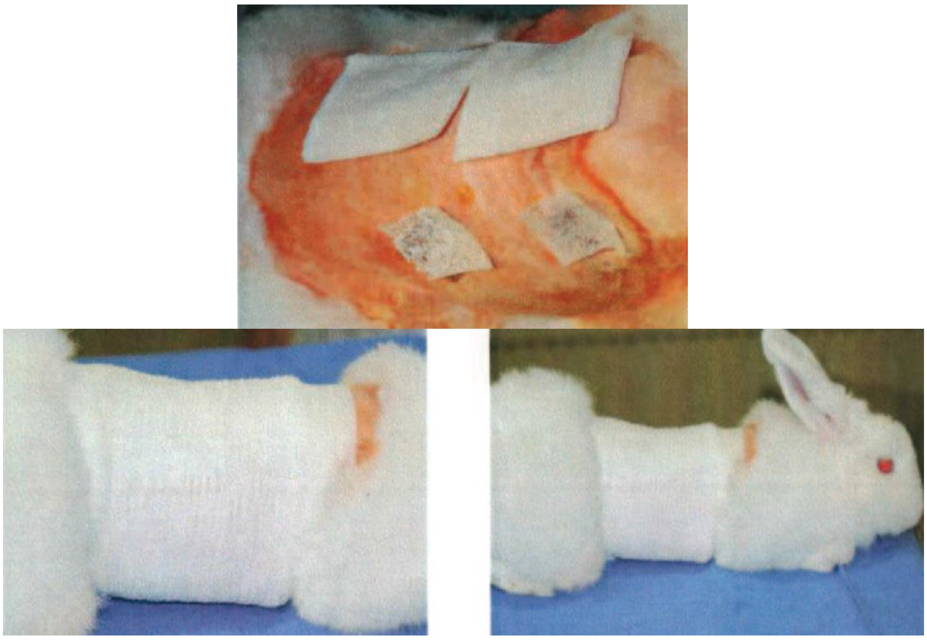

Figure S3. Wound supplying with Medisorb R and gauze control dressing (top panel). Wound supplying with the knitted band (bottom panel).

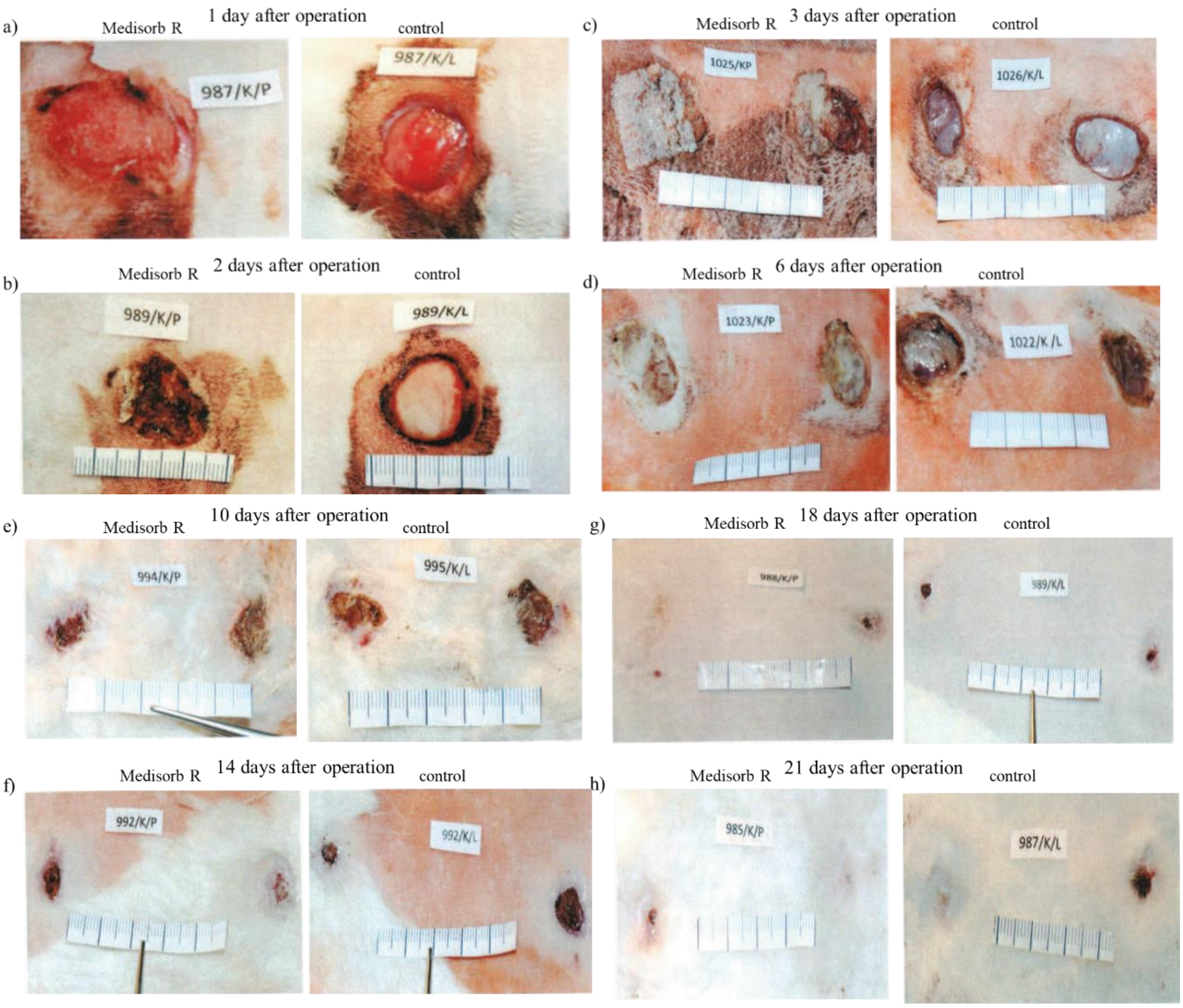

Figure S4. Macroscopic images of operational skin loss in rabbits, 1, 2, 3,6, 10, 14, 18 and 21 days after operation. The wounds were treated with Medisorb R dressing and gauze dressing as control.
